# Deubiquitinase JOSD1 tempers hepatic proteotoxicity

**DOI:** 10.1038/s41420-024-02177-y

**Published:** 2024-09-16

**Authors:** Saheli Chowdhury, Abhishek Sen, Debajyoti Das, Partha Chakrabarti

**Affiliations:** 1 Division of Cell Biology and Physiology, CSIR-Indian Institute of Chemical Biology, Kolkata, India; 2 Academy of Scientific and Innovative Research (AcSIR), Ghaziabad, 201002 India; 3 Present Address: Department of Medicine-Division of Digestive Diseases, David Geffen School of Medicine, University of California, Los Angeles, CA USA

**Keywords:** Apoptosis, Metabolic syndrome

## Abstract

Derangements in protein homeostasis and associated proteotoxicity mark acute, chronic, and drug-induced hepatocellular injury. Metabolic dysfunction-associated proteasomal inhibition and the use of proteasome inhibitors often underlie such pathological hepatic proteotoxicity. In this study, we sought to identify a candidate deubiquitinating enzyme (DUB) responsible for reversing the proteotoxic damage. To this end, we performed a siRNA screening wherein 96 DUBs were individually knocked down in HepG2 cells under proteasomal inhibitor-induced stress for dual readouts, apoptosis, and cell viability. Among the putative hits, we chose JOSD1, a member of the Machado-Josephin family of DUBs that reciprocally increased cell viability and decreased cell death under proteotoxicity. JOSD1-mediated mitigation of proteotoxicity was further validated in primary mouse hepatocytes by gain and loss of function studies. Marked plasma membrane accumulation of monoubiquitinated JOSD1 in proteotoxic conditions is a prerequisite for its protective role, while the enzymatically inactive JOSD1 C36A mutant was conversely polyubiquitinated, does not have membrane localisation and fails to reverse proteotoxicity. Mechanistically, JOSD1 physically interacts with the suppressor of cytokine signalling 1 (SOCS1), deubiquitinates it and enhances its stability under proteotoxic stress. Indeed, SOCS1 expression is necessary and sufficient for the hepatoprotective function of JOSD1 under proteasomal inhibition. In vivo, adenovirus-mediated ectopic expression or depletion of JOSD1 in mice liver respectively protects or aggravates hepatic injury when challenged with proteasome blocker Bortezomib. Our study thus unveils JOSD1 as a potential candidate for ameliorating hepatocellular damage in liver diseases.

## Introduction

The liver is the major protein metabolic hub, and dysregulated hepatic protein turnover could adversely incriminate a range of liver diseases. Accumulation of ubiquitinated proteins and appearance of classic Mallory Denk Bodies in liver histology across many chronic liver diseases like ASH, NASH, Wilson’s disease, HCC, etc. support the conspicuous involvement of altered protein homoeostasis in these conditions [1]. Moreover, disruptions in protein homoeostasis cause ER stress and give rise to hepatic insulin resistance associated with diminished proteasome activity [2]. There is a remarkable accumulation of ubiquitin with a complimentary drop in the proteasome activity in livers of high fat diet fed mice. A subset of human NASH patients also show genetic signatures consistent with reduced proteasome activity [3]. Few proteasomal inhibitors, including Bortezomib, are used widely in chemotherapy in multiple myeloma patients with observable but tolerable hepatotoxicity [4, 5]. Clinical cases are quite prevalent wherein multiple myeloma patients treated with Bortezomib display significant hepatocellular damage and injury [6–8].

The ubiquitin-proteasome system comprising of Ubiquitin, E1-Ubiquitin Activating Enzyme, E2-Ubiquiting Conjugating Enzyme, E3 Ubiquitin Ligase, 26S proteasome and Deubiquitinating Enzymes (DUBs) constitute major cellular proteolytic machinery. By removing ubiquitin from the cognate substrates, DUBs curtail undue or excessive protein degradation [9], turn off or turn on a signalling pathway by proofreading cellular ubiquitin adducts, protect activated ubiquitin from nucleophilic attack [10], liberate unanchored ubiquitin chains from 26S  proteasome and recycle the cellular ubiquitin pool. Humans harbour roughly around 100 DUBs [11, 12], and they are divided into five different families, four of which are cysteine proteases, and the fifth family constitutes zinc-dependent metalloproteases. Ubiquitin-specific processing proteases (USP or UBP in yeast), the ubiquitin C-terminal hydrolases (UCH), ovarian tumour-related proteases (OTU), and the Josephin/Machado–Joseph disease proteases (MJD) constitute the cysteine protease DUB family while JAB1/MPN/Mov34 metalloenzyme (JAMM) comprise the zinc-dependent metalloprotease DUB family [11]. USP, UCH, OTU and MJD families have conserved cysteine and histidine boxes with Cys, His and Asp as the residues in the active site catalytic triad, whereas the JAMM family contains two conserved His and Asp residues coordinated to a Zn residue in its active site [13]. Certain DUBs are specific for selected types of ubiquitination, while others exhibit a broad spectrum of action. The role of DUBs in chronic liver diseases is recently being unravelled. Cylindromatosis (CYLD) and TNFAIP3 are DUBs which have been implicated in hepatic apoptosis, inflammation and fibrosis in the context of non-alcoholic fatty liver disease and hepatocellular carcinoma [14–16]. Also, USP18, when overexpressed specifically in HFD-fed mice livers, could improve hepatic steatosis and insulin sensitivity through deubiquitinating TAK1 [17]. USP2 is known to regulate hepatic gluconeogenesis and glucose metabolism by modulating the hepatic expression of 11β-hydroxysteroid dehydrogenase1 [18].

The cysteine protease JOSD1 belongs to the MJD family of DUBs that primarily cleaves K48 ubiquitin chains when it is monoubiquitinated and has its catalytic active site at C36 [19, 20]. JOSD1 is known to localise to the plasma membrane and interact with the actin cytoskeleton to regulate membrane dynamics and cell motility and influence endocytosis [19]. JOSD1 can inhibit the IFN-1-induced signalling pathway and antiviral response by physically interacting with SOCS1 and enhancing SOCS1 stability by cleaving K48-ubiquitin chains from SOCS1 [21]. JOSD1 could also deubiquitinate MCL1 and stabilise MCL1 to suppress mitochondrial apoptosis and confer chemoresistance in gynaecological cancer [20]. The expression of JOSD1 was aberrant in HNSCC specimens, and its depletion improved cisplastin-induced apoptosis of HNSCC cells, suppressed tumour growth and improved chemosensitivity in vitro via regulation by the epigenetic regulator, BRD4 [22]. JOSD1 deubiquitinates and stabilises mutant JAK2 (JAK2-V617F), and therefore, inactivation of JOSD1 via small molecules led to the degradation of mutant JAK2 in MPNs providing a novel therapeutic approach for the disease [23]. JOSD1 was overexpressed in lung adenocarcinoma (LUAD) tissues, and its knockdown suppressed tumour cell proliferation and inhibited metastasis. It was seen that JOSD1 could deubiquitinate and stabilise Snail protein which promoted epithelial to mesenchymal transition (EMT) in LUAD [24]. In Duck Tembesu virus infection, JOSD1 interacts with SOCS1 via its SH2 domain and stabilises SOCS1. SOCS1, in turn, acts as an E3 ubiquitin ligase, mediating the degradation of IRF7 and inhibiting type I interferon production, leading to the proliferation of the virus [25]. MCL-1 stabilisation by JOSD1 has also been implicated in conferring radioresistance in oral squamous cell carcinoma (OSCC) wherein TRAF4 mediates MCL-1 phosphorylation, making it inaccessible for JOSDI interaction [26]. However, the role of JOSD1 in liver diseases, particularly in proteotoxicity, is unknown.

In this study, we identified JOSD1 as a hitherto unknown candidate for mitigating and combating hepatic proteotoxicity. Using various in vitro and in vivo models of hepatic proteotoxicity, we showed that JOSD1-mediated deubiquitination and stabilisation of SOCS1 is the putative molecular basis of hepatoprotection. Thus, we discovered a novel role of JOSD1 in ameliorating hepatic apoptosis under conditions of proteotoxicity.

## Results

### JOSD1 identified as a crucial regulator of cell death under proteotoxic stress

**Fig. 1:**
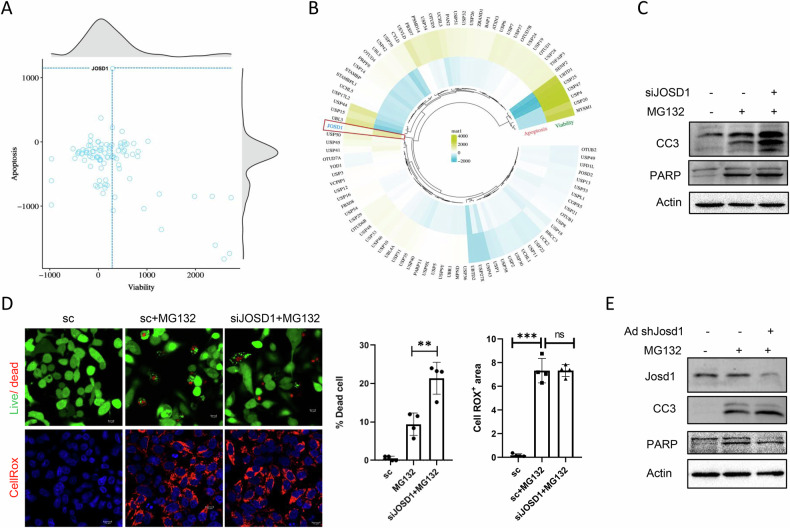
siRNA screening of DUBs and validation of JOSD1. **A** 2D scatterplot for the siRNA screening representing relative luminescence (RLU) as a function of apoptosis in the *y*-axis, whereas the *x*-axis indicates relative fluorescence intensity (RFU) as a measure of cell viability. The distribution density for both measures was plotted against the *x*- and *y*-axis border. Each point represents a normalised relative intensity from three independent experimental replicates. The dotted line (Blue) indicates the respective RFU and RLU values from the JOSD1 knockdown. **B** Circular heatmap representing differential clustering in Euclidean distance matrix using the quantitative measures of viability and apoptosis from the siRNA screening. Euclidean clustering groups RFU and RLU values into clusters, such that the losses of function for individual genes resulting in response are clustered together. **C** JOSD1 knockdown by siRNA transfection in HepG2 cells treated with 5μM MG132 for 16 h and analysed for cell death markers. Cells transfected with scrambled siRNA represent the control in the first lane. **D** (Top) Live Dead Assay in JOSD1 knocked down cells under proteotoxic stress induced with 5μM MG132 for 16 h. Green indicates live cells, whereas red dots indicate dead cells. (Bottom) CellRox assay was performed in control and JOSD1-depleted cells in proteotoxic conditions. Red indicates the level of generated cell ROS. (Right) Statistical analysis of the percentage of dead cells and CellRox^+^ area. *n* = 4 fields/group. Values are presented as mean ± SEM. **P < 0.01, ***P < 0.001. ns not significant. Scale bar—10 µM. **E** JOSD1 was knocked down in primary mouse hepatocytes by adenovirus-mediated delivery and checked for apoptosis markers after induction of proteotoxicity by 5μM MG132 for 16 h. Cells infected with adenovirus carrying scrambled shRNA represent the control in the first lane.

Perturbation in protein turnover and proteostasis via the ubiquitin-proteasome pathway causes significant hepatocellular damage. Here we sought to identify the DUBs responsible for this phenomenon. For this, we used a 96 DUB siRNA library and transfected HepG2 cells with the respective siRNAs followed by proteasome inhibitor MG132 treatment for 16 h. Cell death via caspase 3/7 activation and cell viability were assessed as two antipodal functional readouts (*r* = −0.53, *p* = 0.0001; Supplementary Fig 1A). While the majority of the DUBs did not have a noticeable impact on cellular death or viability (Fig. 1A), we find a cluster of genes, knockdown which resulted in high viability and low apoptosis, indicating the causal candidates (*MYSM1*, *USP20*, *USP4*, *USP47*, *USP25*, and UBTD1) facilitating hepatic proteasomal stress (Fig. 1B). In contrast, we found that the depletion of *JOSD1* resulted in a significant increase in apoptosis with concomitant loss of viability suggesting its crucial importance in protecting against proteotoxic hepatocellular injury (Fig. 1A, B). To validate JOSD1 as a pivotal player in modulating cellular apoptosis under proteotoxic stress, we knocked down JOSD1 using siRNA in HepG2 cells and proteotoxicity was induced with MG132 treatment. Downregulation of JOSD1 did not alter the cellular ubiquitylated protein profile (Supplementary Fig. 1B) but caused an increase in the expression of cleaved caspase 3 (CC3) and cleaved PARP (Fig. 1C) with a significantly elevated number of dead cells (Fig. 1D, top panel). However, there was no change in the level of reactive oxygen species in JOSD1-depleted cells compared to control under proteasomal inhibition, indicating that JOSD1 operates in a ROS-independent pathway (Fig. 1D, bottom panel). A similar observation was seen in primary mouse hepatocytes where adenovirus-mediated knockdown of JOSD1 caused a robust increase in cell death as evidenced by increased expression of CC3 and cleaved PARP when subjected to proteasomal inhibition (Fig. 1E).

### JOSD1 confers protection against cell death in proteotoxic conditions

**Fig. 2:**
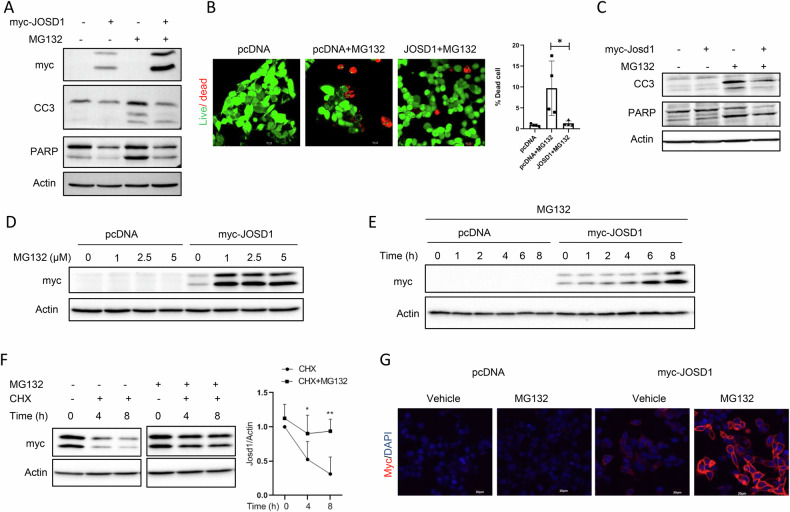
Impact of proteasomal inhibition in HepG2 cells stably expressing JOSD1. **A** Status of cellular apoptosis in JOSD1 overexpressing cell line under proteotoxic stress induced by 5μM MG132 for 16 h. **B** Live Dead Assay in JOSD1 overexpression cell line under proteotoxic condition. Green represents live cells, and red represents dead cells. (Right) Statistical analysis of the percentage of dead cells. *n* = 4 fields/group. Values are presented as mean ± SEM. *P < 0.05, Scale bar—10 µM. **C** JOSD1 overexpressed in primary mouse hepatocytes with the aid of adenovirus-mediated delivery and checked for apoptosis markers under proteotoxic stress. **D** Accumulation of JOSD1 with increasing dose of MG132 for 16 h in JOSD1 overexpression cell line. **E** Accumulation of JOSD1 over time in JOSD1 overexpressing cell line. **F** Cycloheximide chase assay performed under proteotoxic stress. **G** Immunofluorescence in JOSD1 overexpressing cell line with anti-myc tag antibody showing JOSD1 cellular localisation under proteotoxic stress induced with 5μM MG132 for 16 h.

Since the absence of JOSD1 caused significant enhancement of apoptosis, we proceeded to address how JOSD1 confers protection against proteotoxic stress. To this end, we made a HepG2 cell line stably expressing myc epitope-tagged JOSD1 for the next set of experiments. In contrast to the depletion of JOSD1, we observed that expression of JOSD1 caused a noticeable reduction in cellular apoptosis as evidenced by decreased expression of CC3 and cleaved PARP (Fig. 2A) and in the number of dead cells (Fig. 2B). Consistently, adenovirus-mediated overexpression of JOSD1 in primary mouse hepatocytes caused a marked decline in cell death under proteotoxicity (Fig. 2C). Therefore, these observations serve to affirm that JOSD1 indeed rescues hepatocytes from apoptosis under proteotoxic conditions. Interestingly, we observed an accumulation of JOSD1 proteins following MG132 treatment, and such accumulation was found to be dependent both on treatment duration (Fig. 2D) as well as with the dose of MG132 (Fig. 2E). MG132-mediated JOSD1 expression was however not driven by enhanced transcription (Data not shown), but was due to an increase in the half-life of JOSD1 in presence of MG132 as shown by cycloheximide chase assay (Fig. 2F). As ubiquitinated JOSD1 could localise to the plasma membrane to influence membrane dynamics, cell motility, and pinocytosis [19], we sought to investigate if similar translocation and localisation of JOSD1 could be seen under proteasomal inhibition. Immunofluorescence with anti-myc tag antibody showed that JOSD1 was unequivocally accumulated around the cell membrane upon proteasomal inhibition (Fig. 2G). Thus, when hepatocytes are exposed to proteotoxic stress, JOSD1 protein is stabilised, accumulated around the plasma membrane and exercises its anti-apoptotic function.

### Enzymatically inactive JOSD1 C36A mutant fails to rescue proteotoxicity

**Fig. 3:**
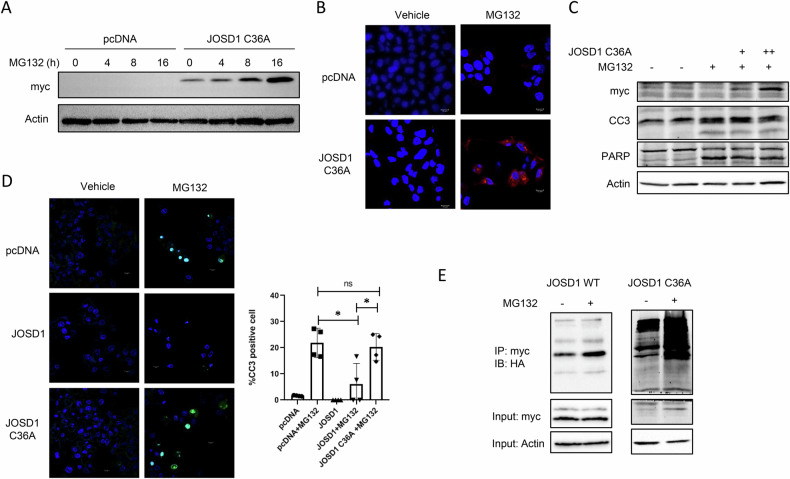
Hepatoprotective effects of JOSD1 C36A mutant during proteotoxicity. **A** Accumulation of JOSD1 mutant protein over time with 5μM MG132 in JOSD1 C36A mutant overexpressing cell line. **B** Cellular localisation of JOSD1 C36A mutant revealed by immunofluorescence with anti-myc tag antibody in JOSD1 C36A mutant overexpressing cell line under proteotoxic stress. **C** Status of hepatic apoptosis in JOSD1 C36A mutant overexpressing cell line under conditions of proteotoxicity. **D** (Left) Live caspase activity shown with caspase substrate activity assay in control, JOSD1 WT and JOSD1 mutant overexpressing cell lines during proteotoxicity induced with 5μM MG132 for 16 h. (Right) Statistical analysis of percentage of cleaved caspase 3 (CC3) positive cells. *n* = 4 fields/group. Values are presented as mean ± SEM. *P < 0.05. ns not significant. Scale bar—10 µM. **E** HepG2 cells stably expressing myc-tagged wild-type JOSD1 or myc-tagged C36A JOSD1 mutant were transfected with HA-tagged ubiquitin. Following MG132 treatment for 4 h, JOSD1 was immunoprecipitated with myc-tag antibody and probed with HA-tag antibody.

JOSD1 has a cysteine residue at the 36th position in its active site [20], and to render the enzyme catalytically inactive, the cysteine was mutated to alanine via site-directed mutagenesis. We next made a JOSD1 C36A mutant overexpressing HepG2 cell line. Upon proteasomal inhibition, the catalytically inactive enzyme also accumulated over time (Fig. 3A), but in contrast to the wild type, it was diffusely localised in the cytoplasm (Fig. 3B) and failed to mitigate cellular apoptosis (Fig. 3C and Supplementary Fig. 2A, B). To further establish the requirement of enzymatic activity in protecting against proteotoxicity, we performed a caspase 3/7 substrate assay, a readout of cell death. Expectedly the caspase 3/7 activity was significantly diminished in JOSD1 overexpressing cells, the activity was restored to that of control cells in the JOSD1 C36A mutant cell line under conditions of proteotoxicity (Fig. 3D). Since JOSD1 is active when it is monoubiquitinated [19], we next checked for the difference in the ubiquitination status of wild type JOSD1 and JOSD1 C36A under proteotoxic stress. We found a remarkable difference in the ubiquitination status of the two proteins wherein wild-type JOSD1 was predominantly monoubiquitinated and JOSD1 C36A was polyubiquitinated under proteotoxicity (Fig. 3E). This observable alteration in the ubiquitination status provides a plausible explanation to the mutant enzyme’s deterred ability to actively exercise protection from cellular apoptosis compared to the wild type protein.

### JOSD1 interacts with and deubiquitinates SOCS1 to mitigate apoptosis under proteotoxic stress

**Fig. 4:**
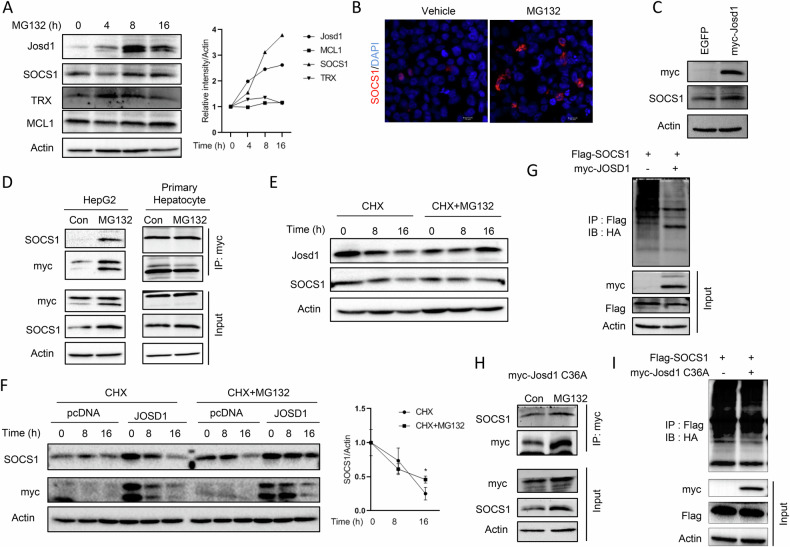
JOSD1 binds, deubiquitinates and stabilises SOCS1 in hepatocytes. **A** (Left) Primary mouse hepatocytes were treated with 5μM MG132 for indicated time periods and checked for JOSD1, SOCS1, TRX and MCL1 protein levels. (Right) Relative band intensities of the corresponding proteins from three experimental replicates. **B** Immunofluorescence staining of SOCS1 in HepG2 cells treated with MG132. **C** SOCS1 protein expression levels in primary mouse hepatocytes overexpressing JOSD1 by adenovirus-mediated method when induced with 5μM MG132 for 16 h. **D** Co-Immunoprecipitation of SOCS1 and JOSD1 in JOSD1 overexpressing cells (left) and primary mouse hepatocytes expressing JOSD1 (right) induced with 5μM MG132 for 4 h. **E** Cycloheximide (CHX) chase assay of JOSD1 and SOCS1 in primary mouse hepatocytes under proteotoxic stress. **F** (Left) Cycloheximide (CHX) chase assay of SOCS1 in JOSD1 overexpressing HepG2 cell line under proteotoxic stress. (Right) Densitometry of band intensities representing half-life of SOCS1 in JOSD1. **G** HepG2 cells stably expressing myc-JOSD1 were transfected with Flag-SOCS1 and HA-ubiquitin and immunoprecipitated with anti-Flag antibody following MG132 treatment for 4 h. **H** Co-immunoprecipitation of endogenous SOCS1 and JOSD1 in JOSD1 C36A mutant overexpressing cells when induced with 5μM MG132 for 4 h. **I** HepG2 cells stably expressing myc-JOSD1 C36A mutant were transfected with Flag-SOCS1 and HA-ubiquitin and immunoprecipitated with anti-flag antibody following MG132 treatment for 4 h. Ubiquitination status of SOCS1 shown upon treatment with 5μM MG132 for 4 h.

To decipher how JOSD1 imparts a protective response in proteotoxicity, we sought for its binding partners, such as SOCS1 and MCL1 [20, 21]. Primary hepatocytes treated with MG132 for 16 h revealed an accumulation of JOSD1 and SOCS1 while TRX and MCL1 did not show much increase (Fig. 4A) There was a noticeable accumulation of SOCS1 in MG132 treated cells (Fig. 4B). Since JOSD1 interacts with and deubiquitinates SOCS1 to negatively regulate type I interferon antiviral activity [21], we sought to identify similar interactions in hepatocytes. Adenovirus-mediated ectopic expression of JOSD1 showed a modest increase of SOCS1 levels in primary hepatocytes (Fig. 4C). Co-immunoprecipitation assay further shows that endogenous SOCS1 interacts with ectopically expressed JOSD1 both in HepG2 and primary mouse hepatocytes (Fig. 4D). Interestingly, such interaction is pronounced under proteotoxic condition when expressions of both JOSD1 and SOCS1 are enhanced. Since MG132-mediated enhanced SOCS1 expression was, however, not transcriptionally regulated (Data not shown), we sought for its protein stability. The half-life of SOCS1 was similarly prolonged in primary mouse hepatocytes in a manner similar to JOSD1 under MG132 treatment (Fig. 4E). Moreover, JOSD1 could enhance the half-life of SOCS1 and protect it from cycloheximide-mediated decrease with time in HepG2 cells under proteotoxic stress (Fig. 4F). Since JOSD1 is a deubiquitinating enzyme, it is expected to deubiquitinate SOCS1 and thus stabilise it. It was indeed seen that JOSD1 could remove ubiquitin chains from SOCS1 and stabilise the protein (Fig. 4G). Though the JOSD1 C36A mutant is also able to bind SOCS1 (Fig. 4H), it does not possess the capability to deubiquitinate SOCS1 under conditions of proteotoxic stress (Fig. 4I).

### JOSD1 is dependent on SOCS1 to mediate its anti-apoptotic property

**Fig. 5:**
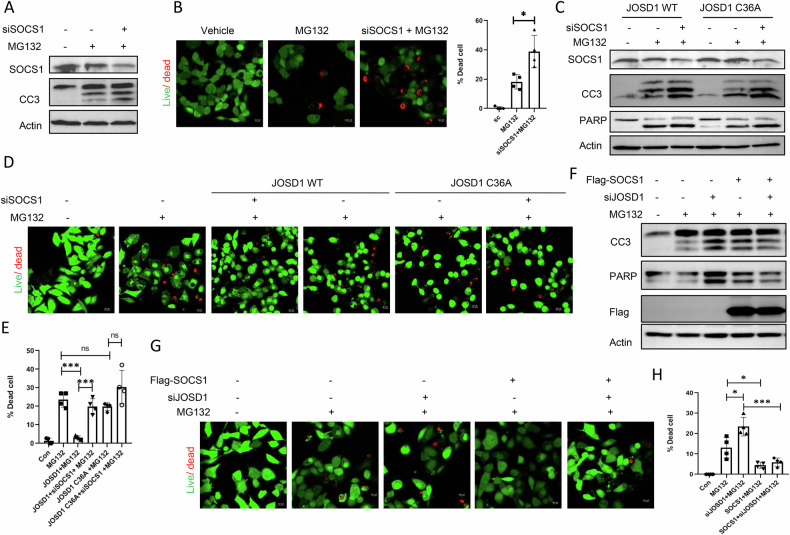
Impact of SOCS1 knockdown and overexpression on the hepatoprotective effects of JOSD1 during proteotoxic stress. **A** Expression levels of apoptotic markers in SOCS1 depleted (siSOCS1) HepG2 cells under proteotoxicity induced with 5μM MG132 for 16 h. **B** (left) Live Dead Assay in SOCS1 depleted HepG2 cells under proteotoxic conditions. Green represents live cells, and red represents dead cells. (Right) Statistical analysis of the percentage of dead cells. *n* = 4 fields/group. Values are presented as mean ± SEM. *P < 0.05, ns not significant. Scale bar—10 µM. **C** Status of expression of apoptotic markers in SOCS1 depleted HepG2 cells stably expressing wild type JOSD1 and JOSD1 C36A mutant under proteotoxicity. **D**, **E** Live Dead Assay in SOCS1 depleted cells stably expressing wild type JOSD1 and JOSD1 C36A mutant under proteotoxic condition. **E** Statistical analysis of percentage of dead cells for (**D**). *n* = 4 fields/group. Values are presented as mean ± SEM. ***P < 0.001. ns not significant. Scale bar—10 µM. **F** HepG2 cells were transfected with flag-tagged SOCS1 and depleted with JOSD1 (siJOSD1), and levels of apoptosis markers were determined by immunoblot. **G**, **H** Live Dead Assay in JOSD1 depleted and SOCS1 expressing HepG2 cells under proteotoxic condition triggered by 5μM MG132 for 16 h. Green represents live cells, and red represents dead cells. **H** Statistical analysis of percentage of dead cells for (**G**). *n* = 4 fields/group. Values are presented as mean ± SEM. *P < 0.05, ***P < 0.001. ns not significant. Scale bar—10 µM.

Since JOSD1 deubiquitinates and stabilises SOCS1, we next examined whether SOCS1 alone could impede proteotoxicity in hepatocytes. We found that overexpression of SOCS1 caused a decrease in CC3 expression and number of dead cells (Supplementary Fig 3A–C), while its downregulation conversely caused an increase in CC3 expression (Fig. 5A) and number of dead cells (Fig. 5B) under proteasomal inhibition in a manner similar to JOSD1.

Next, we sought to determine whether SOCS1 sufficiency was a prerequisite for JOSD1’s ability to modulate cell death under proteotoxic conditions. To this end, SOCS1 was knocked down in both wild-type and mutant JOSD1 overexpressing cells. The absence of SOCS1 attenuated JOSD1’s ability to reverse apoptosis under proteotoxic stress, while JOSD1 C36A cells were unaffected (Fig. 5C–E). Conversely, overexpression of SOCS1 in JOSD1-depleted cells rescued hepatic cells from undergoing enhanced apoptosis subjected to proteotoxicity (Fig. 5F–H). These observations thereby establish SOCS1 as a necessary and sufficient downstream effector that aids JOSD1 in shielding hepatocytes from unregulated apoptosis under proteotoxic stress.

### JOSD1 ameliorates apoptosis in the liver under proteotoxicity

**Fig. 6:**
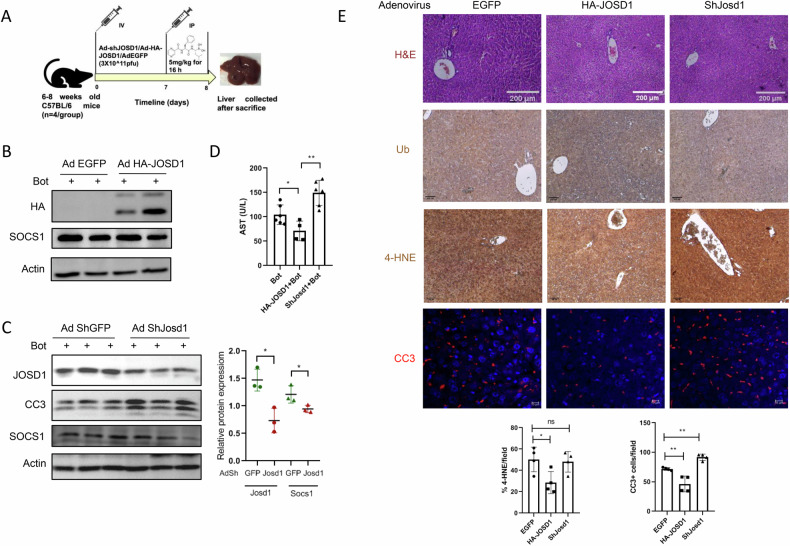
Depletion and ectopic expression of JOSD1 in mouse liver under bortezomib treatment. **A** Graphical representation of in vivo model and experimental scheme. **B** Overexpression of JOSD1 by intravenous injection of 3 × 10^11^ pfu Ad HA-JOSD1 and status of SOCS1 expression in mouse liver following intraperitoneal administration of 5 mg/kg of bortezomib for 16 h. The control group were injected with 3 × 10^11^ pfu Ad EGFP. **C**. (Left) JOSD1 gene was depleted by administering 3 × 10^11^ pfu Ad shJOSD1 and proteotoxicity was induced by bortezomib. (Right) Statistical analysis showing densitometric analysis of JOSD1 and SOCS1 protein expressions. *n* = 3 animals/group. Values are presented as mean ± SEM. *P < 0.05, **P < 0.01. ns not significant. **D** Plasma levels of AST in control, JOSD1 knockdown and JOSD1 overexpression murine models of acute proteotoxicity. **E** (Top Panels) Liver tissue sections from EGFP, HA-JOSD1 and shJOSD1 carrying adenovirus injected mouse stained with H&E for tissue architecture, immunostained with ubiquitin antibody (Ub), 4-hydroxynonenal (4-HNE) and cleaved caspase 3 (CC3). (Bottom Panels) Statistical analysis of 4-HNE positive area and number of CC3 positive cells across the three groups of animals. *n* = 4 animals/group. Values are presented as mean ± SEM. *P < 0.05, **P < 0.01. ns not significant.

We next examined whether JOSD1 could temper hepatic proteotoxicity in an in vivo murine model subjected to acute proteasomal inhibition. To this end, we either knocked down or overexpressed JOSD1 in the livers of mice via adenovirus-mediated delivery by tail vein injection. After 7 days, the mice were intraperitoneally injected with 5 mg/kg of bortezomib for 16 h following which they were sacrificed (Fig. 6A). Although overexpression of JOSD1 did not alter SOCS1 protein levels (Fig. 6B), we find a decline in SOCS1 following knockdown of JOSD1 (Fig. 6C). Interestingly, the levels of plasma AST, a liver enzyme which serves as a marker for liver injury, was reciprocally increased upon JOSD1 knockdown and decreased by overexpression of JOSD1 (Fig. 6D). However, there were no remarkable changes in liver tissue architecture (H&E), levels of oxidative stress (4-HNE) and ubiquitination status across the three groups (Fig. 6E). Notably, knockdown of JOSD1 exacerbated apoptosis as evidenced by increased expression of CC3 both in western blot (Fig. 6C) and immunofluorescence assays (Fig. 6E). In contrast, ectopic expressions of JOSD1 substantially assuaged hepatic injury by lessening cell death compared to mice injected with vehicle controls (Fig. 6E). Taken together, JOSD1 serves as a potent hepatoprotective enzyme in vivo under proteotoxic stress.

## Discussions

**Fig. 7:**
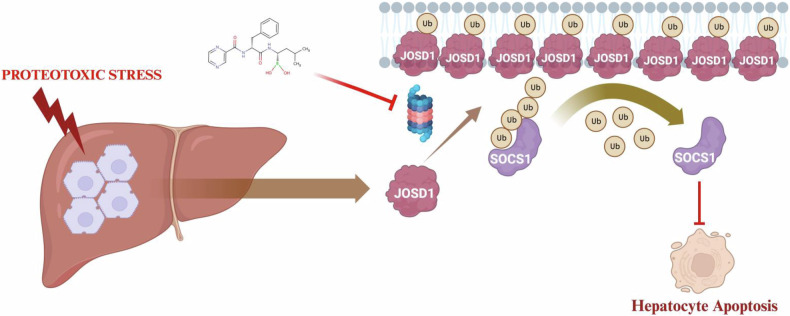
Schematic of the hepatoprotective mechanism of JOSD1.

The roles and functional implications of DUBs in various cellular events are recently being unravelled. Although accumulating evidence suggests the involvement of DUBs in steatotic liver diseases, very few studies so far have elucidated the involvement of DUBs in hepatic manifestations of xenobiotic toxicity. Since the liver is one of the most proteostatically challenged organs and there are compelling evidence of marked aberrations in protein homoeostasis culminating in extensive hepatic injury [2, 3], involvement of UPS and its components in such contexts are unknown. In this study, we explored the roles of DUBs in proteasomal inhibitor-induced models of hepatocellular injury. A first-of-its-kind siRNA screening assay identified JOSD1 as a candidate DUB that reversed hepatic proteotoxicity. We have further identified the downstream molecular event of deubiquitination of SOCS1 through which JOSD1 elicits the protective response. Loss and gain of function studies further established its potential hepatoprotective effects in the in vivo setting (Fig. 7).

Under proteotoxic stress, hepatocellular JOSD1 revealed unique molecular features such as monoubiquitination, accumulation and plasma membrane localisation, which are consistent with the existing literature from different cellular contexts [19, 20]. Interestingly, the catalytically inactive JOSD1 mutant was not only ineffective against proteotoxicity but also did not exhibit such molecular events. However, how exactly monoubiquitinated and membrane-bound JOSD1 elicits its protective response needs further investigation. Our study opens a plethora of avenues to consider the question of the therapeutic scope and viability of JOSD1 during periods of dysfunctional protein homoeostasis. It also needs to be seen how JOSD1 is implicated in chronic liver diseases where there is extensive compromised liver function, hepatic injury and deranged proteostasis.

Towards identifying the downstream JOSD1 effecter responsible for its hepatoprotective effects, the study of existing JOSD1 substrates revealed analogous accumulation of SOCS1 under proteotoxic stress. Moreover, SOCS1 also prevents cellular death under proteotoxicity and is sufficient for the protective response of JOSD1. As a negative regulator of type 1 interferon signalling and inflammation, SOCS1 has been shown to modulate cell death in cancer and in acute stress [27, 28]. Conversely, overexpression of SOCS1 could augment hepatic steatosis and insulin resistance [29]. SOCS1 at the intersection of metaflammation and infection-induced inflammation could thereby balance a tissue-specific disease outcome. Beyond the consensus anti-inflammatory function, the present study thus describes a new functional role of SOCS1 in the context of hepatic proteotoxicity.

Our present work has few limitations. For example, the sufficiency of SOCS1 for the anti-apoptotic function of JOSD1 needs further validation in the in vivo setting. The downstream effector of SOCS1 is also not known. The differential impact of other anti-apoptotic JOSD1 substrates under proteotoxicity needs further studies. Also, the functional role of the JOSD1–SOCS1 axis in human subjects is to be investigated.

In summary, we have substantially unearthed that JOSD1 bestows hepatocytes with a protective armour to shelter cells from apoptosis by increased expression and localisation around the plasma membrane, ultimately culminating in deubiquitination and stabilising SOCS1. Our data uniquely integrates and assimilates the little antecedent knowledge about the mechanisms of JOSD1 in various cellular events into one consolidated form while also unravelling an intricate involvement of JOSD1 in a previously unknown cellular event, hepatic apoptosis and hepatic injury under conditions of proteotoxic stress.

## Materials and methods

### Animal study

Protocols for animal experiments were approved by the Institutional Animal Ethics Committee at CSIR-IICB under the aegis of the Committee for Control and Supervision of Experiments on Animals (CPCSEA), Ministry of Environment and Forest, Government of India. Six to eight-week-old C57BL/6 male mice were divided into three groups (n = 6/group). One group was injected with 3 × 10^11^ pfu AdEGFP and the other two with 3 × 10^11^ pfu shJOSD1 and 3 × 10^11^ pfu AdJOSD1, respectively, via tail vein. After a week, each group was intraperitoneally injected with 5 mg/kg of Bortezomib (Merck, MA, USA) and sacrificed after 16 h. Serum was collected for liver enzyme analysis. Liver sections were collected in formalin (for histological analysis) and lysis buffer (for protein lysate preparation for Western Blot).

### AST estimation

Aspartate aminotransferase (AST) levels were measured using a commercially available kit (Randox Laboratories, Crumlin, UK) utilising the kinetic method following manufacturer’s protocol in a semi-auto analyser Microlab 300 (Clinical Systems, EliTech Group, Puteaux, France) from serum of sacrificed mice.

### Histology, immunohistochemistry and immunofluorescence of liver tissue sections

Liver tissue sections were processed for H&E, and images were taken using Evos XL Core (Thermo Fisher Scientific, MA, USA).

Liver tissue sections were first deparafinized by heating at 85 °C for 10 min, followed by treatment with decreasing gradients of alcohol. Antigens were retrieved by heating and permeabilising sections with sodium citrate buffer. Immunohistochemistry was then performed using two kits: VECTASTAIN ABC KIT (Biotinylated Horseradish Peroxidase Anti-rabbit IgG) and ImmPRESS Duet Double staining Polymer kit (Horseradish Peroxidase Anti-Mouse IgG/Alkaline Phosphatase Anti-Rabbit IgG) [VectorLabs, CA, USA] following manufacturer’s protocol. Images were taken with Evos XL Core (Thermo Fisher Scientific, MA, USA). Ubiquitin (#58395S, Cell Signalling Technology, MA, USA) and 4-HNE (#MAB3249, R&D Systems, Minneapolis, USA) primary antibodies were used for immunohistochemistry.

For immunofluorescence, a blocking buffer was added to block the non-specific binding of antibodies. Sections were then incubated with Cleaved Caspase 3 primary antibody tagged with AlexaFluor 647 (#9602S, Cell Signalling Technology, MA, USA) at a dilution of 1:100 overnight in a humidified chamber at 4 °C. The next day, sections were stained with 25 µg/ml HOECKST342 (Thermo Fisher Scientific, MA, USA) and viewed under a Leica TCS SP8 STED microscope (Leica Microsystems, Wetzlar, Germany).

### Generation of adenovirus

shJOSD1 carrying adenovirus was generated using BLOCK-iT^TM^ Adenoviral RNAi Expression System (Thermo Fisher Scientific, MA, USA) following manufacturer’s protocol. The following sequences were used to generate the ds oligo of shJOSD1: Top strand oligo-5′ caccgcacaagaagagcatgctgggaaatgggaacgaattcccatttcccagcatgctcttcttgtg 3′; Bottom strand oligo-5′ aaaacacaagaagagcatgctgggaaatgggaattcgttcccatttcccagcatgctcttcttgtgc 3′. Briefly, pENTR clone carrying shJOSD1 ds oligo was obtained and recombined with pDEST. The final product was digested with PacI enzyme and transfected in HEK293A cells to generate viable adenoviral particles. Crude adenoviral HEK293A cell lysate expressing JOSD1 was purchased from ABM (#252540540200) [NY, USA]. Once actively replicating viral particles started to lyse cells, the media, along with adherent cells, were harvested. After repeated cycles of freezing and thawing, active viral particles were purified using Pure Virus^TM^ Adenovirus Purification Kit (Cell Biolabs, CA, USA) as per the manufacturer’s protocol. The viral titre was determined spectrophotometrically, and the purified virus was stored at −80 °C until further use.

### Primary mouse hepatocytes

Primary mouse hepatocytes were isolated and cultured as described previously [30]. Briefly, 8–12 weeks old chow-fed black male mouse (C57bl/6) was sacrificed, portal vein was cannulated, and liver was perfused with 20 ml of HBSS (Hank’s Balanced Salt Solution, without CaCl_2_) by Masterflex digital peristaltic pump (Cole-Parmer, IL, USA). After cutting the inferior vena cava, 25 ml of Collagenase (Roche, Merck, MA, USA) solution (1 mg/ml) in HBSS (containing 1 mM CaCl_2_) was allowed to pass through the liver at a constant flow rate of 3 ml/min. The pieces of digested liver tissue were then minced in HBSS (containing 1 mM CaCl_2_). The resulting suspension was passed through a 100 μm cell strainer (SPL Life Sciences, Pochon, Kyonggi-do, South Korea), and the filtrate was centrifuged at 50*g* for 2 min at 4 °C. The cellular pellet was carefully resuspended in William’s Medium E (1×) [Gibco, Thermo Fisher Scientific, MA, USA] supplemented with 1% antibiotic antimycotic solution (HiMedia, Mumbai, India) for plating and allowed to settle down for 6 h in Collagen (Thermo Fisher Scientific, MA, USA) pre-coated cell culture plates. The adhered hepatocytes were maintained in Hepatocytes Basal Medium with Ultraglutamine1 (Lonza, Basel, Switzerland).

### Cell culture

HepG2 (Human hepatoma cell line, ATCC, Virginia, USA) and HEK293A (Human embryonic kidney cell line, Thermo Fisher Scientific, MA, USA) were cultured in MEM (Eagle’s minimal essential medium) and DMEM (Dulbecco’s Modified Eagle Medium) [HiMedia, Mumbai, India], respectively, supplemented with 10% Foetal Bovine Serum (Gibco, Thermo Fisher Scientific, MA, USA) and 1% antibiotic antimycotic solution containing Penicillin, Streptomycin and Amphotericin [HiMedia, Mumbai, India]. HEK293A cells require an additional 1% non-essential amino acids. HA-Ub plasmid (Addgene, MA, USA), mycDDK-JOSD1 plasmid (# RC201968, Origene, Maryland, USA), and flag-SOCS1 plasmid (#OHu17289, GenScript, NJ, USA) were transfected using Lipofectamine 2000 (Thermo Fisher Scientific, MA, USA) according to manufacturer’s protocol.

### Cell harvesting, protein estimation and preparation

Washed cells were homogenised in a lysis buffer containing a 0.5% protease inhibitor cocktail (Merck Millipore, MA, USA). The resultant slurry was centrifuged at 20,000*g* for 20 min at 4 °C. Protein concentration was estimated using Bradford assay (BioRad, CA, USA). In total, 30–60 μg of protein samples were prepared using 1× Laemmli’s buffer, heated at 95 °C for 10 min, cooled and centrifuged at 12000*g* for 2 min prior to loading into polyacrylamide gel.

### Western Blot

Proteins were resolved in 10% SDS PAGE and transferred to a PVDF membrane (Millipore, MA, USA) having a pore size of 0.45 μm. Membranes were washed in 1× TBST (pH 7.6) containing 1% Tween 20 (Sigma Aldrich, MO, USA), and blocked with 5% skimmed milk powder (HiMedia, Mumbai, India). The required primary antibody (JOSD1 [#ab118221, Abcam, Cambridge, UK], Cleaved Caspase 3 [#9661s], PARP [#9542S], myc-tag [#2278S], HA-tag [#3724S], flag-tag [#14793S] [Cell Signalling Technology, MA, USA], Actin [#A5316, Sigma Aldrich, MO, USA], SOCS1 [A7754], MCL-1 [#A0250] and TRX [#A0053] [Abclonal, MA, USA]) prepared at 1/1000th dilution with 1× TBST containing 1% Bovine Serum Albumin and 0.04% Sodium Azide was added to the membrane and incubated overnight at 4 °C. Next day, the membrane was washed multiple times with 1× TBST to remove any unbound primary antibody and incubated with anti-rabbit (#31460) or anti-mouse secondary antibody(#31430) [Thermo Fisher Scientific, MA, USA] for 1 h at room temperature and washed again for multiple times with 1× TBST. The membrane was then developed using Clarity™ ECL Western Blotting Substrate (BioRad, CA, USA) and viewed in ChemiDoc MP (BioRad, Hercules, CA, USA).

### Site-directed mutagenesis

JOSD1 C36A mutant was generated using QuickChange II Site-Directed Mutagenesis Kit (Agilent, CA, USA) following manufacturer’s protocol. The following primers were designed using the primer design guidelines of the kit: Forward Primer: 5′ cagcgcagggagcttgctgccctccacgccctc 3′; Reverse Primer: 5′ gagggcgtggagggcagcaagctccctgcgctg 3′. PCR amplicons were digested with DpnI enzyme, transformed in DH5α competent cells, plasmids were isolated from selected colonies, and sequenced for confirming the mutation.

### Generation of JOSD1 and JOSD1C36A mutant cell lines

HepG2 cells were transfected with mycDDK-JOSD1 and mycDDK JOSD1 C36A mutant plasmids using Lipofectamine 2000 (Thermo Fisher Scientific, MA, USA). After 48 h, cells were subjected to 100 μg/μl of Gentamycin (InvivoGen, CA, USA) selection, and pooled clones stably expressing the proteins of interest were propagated.

### siRNA

siRNA transfection was carried out using Lipofectamine^TM^ RNAiMax Transfection Reagent (Thermo Fisher Scientific, MA, USA). 72 h post transfection, cells were processed for western blot analysis. Human JOSD1 siRNA SMARTPOOL (Cat No. L-017674-00-0005) and Human SOCS1 siRNA SMARTPOOL (Cat No. L-011511-00-0005) were purchased from Dharmacon, Horizon Discovery (Waterbeach, UK).

### DUB screening

A loss of function screening using Human ON-TARGETplus siRNA Library—Deubiquitinating Enzymes (Horizon Discovery, Waterbeach, UK; Cat No. G-104705) in the HepG2 cell line was designed. HepG2 cells were seeded in 96 well luminescence and fluorescence-compatible plates and transfected with the respective siRNAs for 72 h. Sixteen hours before cell harvesting, proteasomal inhibition was imposed upon the cells with 10 μM of MG132 (Sigma Aldrich, MO, USA). ApoLive-Glo Multiplex Assay Kit (Promega, Wisconsin, USA) as per manufacturer’s protocol. Cell viability was estimated by fluorescence, and caspase 3/7 activity was estimated by luminescence. Two-way normalisation was done with both the viability measurements (% live cells) and Caspase 3/7 activity measurements. For both assays, scrambled siRNA transfected cells treated with MG132 were used as a control group. To avoid accumulating type 1 errors for the experimental replicates, we used normalisation by summing all data points in a replicate. Values were eliminated by checking for outliers. Measures accounted for both viability and apoptosis were plotted as ring cluster plots by using the *circlize* package in R.

### Live dead assay

LIVE/DEAD®Viability/Cytotoxicity Kit for mammalian cells (Thermo Fisher Scientific, MA, USA) was used for staining control as well as JOSD1 overexpressing stable cell line after proteasomal inhibition for 16 h and imaged with Leica TCS SP8 STED microscope.

### Caspase 3/7 substrate activity assay

Cells were seeded in confocal dishes and treated with MG132 for 16 h. The caspase 3/7 substrate activity assay (Thermo Fisher Scientific, MA, USA) reagents were added and incubated as per the manufacturer’s protocol. Cells were then visualised using the Leica TCS SP8 STED microscope.

### Cell Rox assay

Cells were subjected to MG132 treatment for 16 h and generation of cellular reactive oxygen species (ROS) was measured using CellROX^TM^ Deep Red Reagent (Thermo Fisher Scientific, MA, USA) following manufacturer’s protocol. ROS production was imaged using Leica TCS SP8 STED microscope.

### Cycloheximide Chase assay

HepG2 cells or primary mouse hepatocytes were treated with either 50 μg/ml of cycloheximide (Calbiochem, Merck Millipore, MA, USA) alone or in combination with 5 μM of MG132 for required time periods. Cells were then harvested using protein lysis buffers at different time points for performing western blot.

### Immunoprecipitation

In total, 500 μg of crude cell lysate was precleared with 10 μl of PureProteome Protein A Magnetic Beads (Merck Millipore, MA, USA) for 30 min. The supernatant containing the pre-cleared crude cell lysate was collected by attaching the tubes to a Magna Rack (Merck Millipore, MA, USA). This pre-cleared crude cell lysate was then used for an overnight reaction at 4^o^C for immune complex formation with fresh 10 μl of PureProteome Protein A Magnetic Beads and the required amount of primary antibody [anti-myc tag (#05-419, Merck, MA, USA) anti-flag tag (Cell Signalling Technology, MA, USA)]. The next day, immune complexes were retrieved by washing magnetic beads with PBS and 0.1% Triton-X100 thrice to remove non-specific molecules. The eluted immune complex was collected by centrifugation and analysed by western blot.

### Immunofluorescence

Cells were fixed with 4% paraformaldehyde for 15 min, washed with 1× PBS and blocked with a serum of the species in which the secondary antibody was raised and Triton-X100 for 1 h at room temperature. Cells were then incubated with anti-myc antibody (1:200 dilution) overnight at 4 °C followed by incubation with Goat Anti-Rabbit Alexa Fluor 647 (#A21245, Invitrogen, Thermo Fisher Scientific, MA, USA) conjugated secondary antibody at a ratio of 1:250 for 2 h. Cells were counterstained with 25 µg/ml HOECKST342 and visualised with a Leica TCS SP8 STED microscope.

### Image and statistical analysis

Confocal images were analysed in Image J by counting the relevant dots for live or dead cells or caspase-positive cells across control and experimental groups. For each experiment, *n* = 4 fields/groups were taken into consideration for further calculation and analysis. Densitometry was performed by measuring the protein band intensities obtained in western blots using Image J. Obtained values were normalised with actin. All statistical analysis and graphs were done in GraphPad Prism 8.0.1 (San Diego, CA, USA) using *Z* statistics, data are represented as mean ± SEM with *p* value cut off <0.05.

## Supplementary information

uncropped western blots

Supplementary Information

## Data Availability

Data sharing is not applicable to this article as no datasets were generated or analysed during the current study.
